# The importance of individual variation for the interpretation of behavioural studies: ethanol effects vary with basal activity level in zebrafish larvae

**DOI:** 10.1007/s00213-021-05932-6

**Published:** 2021-09-11

**Authors:** Raissa Davis, Floris Luchtenburg, Michael Richardson, Marcel Schaaf, Christian Tudorache, Hans Slabbekoorn

**Affiliations:** Institute of Biology, Leiden University, Leiden, the Netherlands

**Keywords:** Coping style, Personality, Juvenile zebrafish, Behaviour, Ethanol

## Abstract

Standardization and reduction of variation is key to behavioural screening of animal models in toxicological and pharmacological studies. However, individual variation in behavioural and physiological phenotypes remains in each laboratory population and can undermine the understanding of toxicological and pharmaceutical effects and their underlying mechanisms. Here, we used zebrafish (ABTL-strain) larvae to explore individual consistency in activity level and emergence time, across subsequent days of early development (6–8 dpf). We also explored the correlation between these two behavioural parameters. We found inter-individual consistency over time in activity level and emergence time, but we did not find a consistent correlation between these parameters. Subsequently, we investigated the impact of variation in activity level on the effect of a 1% ethanol treatment, suitable for our proof-of-concept case study about whether impact from pharmacological treatments might be affected by inter-individual variation in basal locomotion. The inter-individual consistency over time in activity level did not persist in this test. This was due to the velocity change from before to after exposure, which turned out to be a dynamic individual trait related to basal activity level: low-activity individuals raised their swimming velocity, while high-activity individuals slowed down, yielding diametrically opposite response patterns to ethanol exposure. We therefore argue that inter-individual consistency in basal activity level, already from 6 dpf, is an important factor to take into account and provides a practical measure to improve the power of statistical analyses and the scope for data interpretation from behavioural screening studies.

## Background

Behavioural screening has become an important tool for studies on animal models for medical and pharmaceutical applications (Anisman and Matheson 2005; Belzung and Philippot 2007; Champagne et al. 2010). Traditionally, this field has been dominated by rodents as animal models (Belzung and Griebel 2001; Prut and Belzung 2003; Champagne et al. 2010), while zebrafish (*Danio rerio*) have become a popular alternative over the last decades (Lieschke and Currie 2007; Khan et al. 2017; Fontana et al. 2018; Meshalkina et al. 2018). General advantages of laboratory studies with any species include standardized test conditions and individual test animals of known genetic background and identical experience (Sukoff Rizzo and Silverman 2016). This can reduce between-experiment variation (Beynen et al. 2003; Crabbe et al. 1999; Wahlsten 2001) and also decrease animal use through an increase in test sensitivity (Festing 2004a, b). However, the reduction of environmental variability can, paradoxically, also be problematic for replication of results across laboratories, as standardised conditions may elevate the impact of otherwise subtle housing differences or inter-individual variation among strains (Würbel 2002; Richter et al. 2009). Inter-individual variation may also be critical within strains, and distinct variation in behavioural and physiological phenotypes remains an issue of concern for test populations that are often erroneously assumed to be homogenous (Egan et al. 2009; Araujo-Silva et al. 2018; Demin et al. 2019).

Individuals within natural and laboratory populations can use different physiological and behavioural strategies to adapt and cope with their environmental challenges (Øverli et al. 2007; Dingemanse and Wolf 2010; Conrad et al. 2011; Roy and Bhat 2018). These individually variable strategies have been described in many vertebrates, such as birds, rodents, and fish, and have shown to be consistent over time and across contexts. Such consistent inter-individual variation has been labeled “coping style”, “personality”, “behavioural syndrome”, or “temperament” (Koolhaas et al. 1999; Gosling 2001; Sih et al. 2004; Réale et al. 2007, respectively), and dependent on the sub-discipline, the relative emphasis is on physiology or behaviour or on the inclusion of few or many traits (see MacKay and Haskell 2015). Independent of the label or emphasis, taking individual variation into account can be critical for the interpretation of behavioural screening data. Variation in baseline parameters may, for example, preclude a significant treatment effect at group level and thereby conceal highly relevant but variable effects for different individuals (MacKenzie et al. 2009; Tudorache et al. 2018; Demin et al. 2019).

Consistent inter-individual variation could also be important for the well-studied effect of ethanol on animal behaviour, which has relevance for alcohol-related health issues in humans. Alcohol consumption can cause a wide variety of human diseases, including liver and cardiovascular problems, foetal alcohol spectrum disorders, and psychiatric conditions in adults (Centerwall and Criqui 1978; Stade et al. 2009; Bakoyiannis et al. 2014). The majority of research, using animal models to understand and treat human alcoholism, has historically exploited rats and mice (Bennett et al. 2006; Bell et al. 2017). However, zebrafish have also been discovered as suitable system in this field to study alcohol-related toxicology and addiction (Gerlai et al. 2000; Echevarria et al. 2011), including investigations into alcohol tolerance, sensitization, and withdrawal (Cachat et al. 2010; Tran and Gerlai 2014; Tran et al. 2015), and individual variation in and chronic exposure effects on stress response mechanisms (Wong et al. 2019; Goodman and Wong 2020; Du et al. 2020).

Dose–response patterns and the time course of emerging alcohol effects on zebrafish are available from studies on both adults and larvae. MacPhail et al. (2009), for example, showed increased swimming activity in adult zebrafish exposed to 1% and 2% ethanol, while activity was severely decreased in 4% (also see Irons et al. 2010; de Esch et al. 2012). The stimulating effects for low doses were found for both light and dark conditions, but transition in behaviour after lights were switched on were delayed relative to control groups. The latter was attributed to potentially lowered visual sensitivity, an ethanol-induced effect also reported for zebrafish larvae (Bilotta et al. 2002; Matsui et al. 2006).

Zebrafish larvae in early stages of development (5–8 dpf) exhibit similar concentration-dependent behavioural responsiveness as adult zebrafish in terms of activity level (Lockwood et al. 2004; Ikeda et al. 2013; Puttonen et al. 2013). The general pattern of impact is relatively consistent among studies, despite some variation in the exact concentrations applied (Guo et al. 2015; Tran et al. 2016a; Du et al. 2020). A concentration of 0.5% alcohol typically has a stimulating effect, with elevated swimming velocity and erratic swimming patterns, while 1.0% alcohol has an opposite, sedative effect, as also confirmed in a recent study by Tsang et al. (2019). The detrimental effects of high concentrations are known to emerge gradually, with a biphasic response pattern: an initial upregulation of activity for about 30 min of exposure is followed by a downregulation for another 30 min (Gerlai et al. 2006; Tran and Gerlai 2013b). The up- and downregulation are associated with the gradual and steady rise in ethanol uptake into the brain over time (Gerlai et al. 2009; Rosemberg et al. 2012; Tran et al. 2015; Guo et al. 2015). Various interactive mechanisms have been proposed to play a role in explaining these effects, including an effect on functioning of the HPI axis (Du et al. 2020) and on GABA_A_ receptor expression (Goodman and Wong 2020).

Zebrafish, besides being a suitable model for alcohol effects, are also suitable for studies on intra-specific, mechanistic variation and potential methodological issues with inter-individual variation, as distinct behavioural and physiological phenotypes are well-established (Egan et al. 2009; Oswald et al 2012; Tran and Gerlai 2013a; Tudorache et al. 2013; Roy and Bhat 2018). Responsiveness to a specific alcohol concentration may not only vary among different populations of zebrafish (Dlugos and Rabin 2003; Gerlai et al. 2009), but also among different individuals of the same population (Dlugos et al. 2011; Leite-Ferreira et al. 2019; Wong et al. 2019; Goodman and Wong 2020). Araujo-Silva et al. (2018), for example, showed that bold individuals were not much affected in shoaling and explorative behaviour, but that shy individuals, who tend to shoal more and explore less, became also more explorative with exposure to 0.1 and 0.5% ethyl alcohol (also see Araujo-Silva et al. (2020). This kind of variation in responsiveness among individuals can already apply to the early stages of development (Budaev and Andrew 2009; Tudorache et al. 2014) and, if neglected, can be problematic in the interpretation of data from behavioural screening studies (c.f. MacKenzie et al. 2009; Demin et al. 2019; Leite-Ferreira et al. 2019).

In this study, we used zebrafish larvae as a model in two tests to explore individual consistency across subsequent days of early development (6–8 dpf) and to investigate the impact of consistent individual variation on the effect of alcohol exposure (6 dpf). We aimed to answer the following questions in the first test: (1) Is individual variation in activity level consistent between subsequent days? (2) Is individual variation in emergence time consistent between subsequent days? (3) Is consistent individual variation in activity level correlated to consistent individual variation in emergence time? In the second test, we investigated the activity-level dependent effect of alcohol in order to address the question: (4) Do low- and high-activity zebrafish larvae respond the same to a non-lethal 1% ethanol exposure? The latter test could reveal potential pitfalls in using sample means for the interpretation of behavioural screening studies.

## Methods

### Animals and housing

We used zebrafish larvae from the ABTL-strain bred at Leiden University (c.f. Tudorache et al. 2014; Amin et al. 2016). The ABTL wild-type zebrafish (*Danio rerio*) strain in this study originates from crossbreeding of the AB and Tüpfel Long Fin (TL) strains. Originally obtained from the Hubrecht Laboratory (Utrecht, the Netherlands), this strain was maintained in our laboratory for a minimum ten generations at the time of experimentation. New generations were created by mating of 60–80 individuals (1:1 ratio male to female) from the previous generation. This segregated hybrid line was chosen because of its large genetic variation, with contributions from both original AB and TL strains (Stickney 2002; Guryev 2006; Brown et al. 2012).

**Fig. 1:**
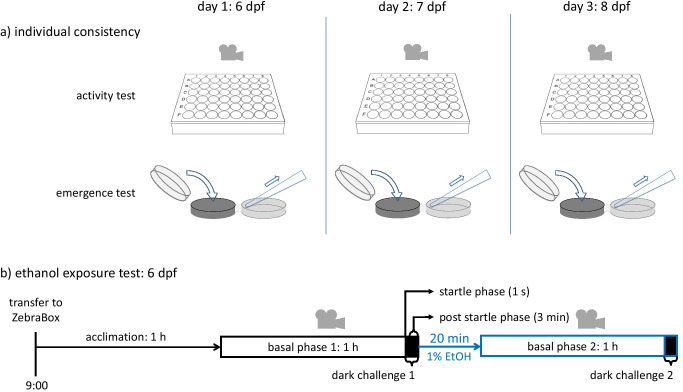
Overview of the two behavioural tests. **a** Individual consistency: schematic representation of the activity test (above), using a 48-well plate, recorded with a top camera view inside the ZebraBox, alongside the three times repeated protocol time line on three subsequent days: 6 dpf, 7 dpf, and 8 dpf; and the emergence test (below): each day, the activity test was followed by an emergence test with the same individual. Three times, we assessed the time it took an individual to emerge. **b** Ethanol test: after an acclimation phase, followed by a 1-h basal phase with lights on, a sudden dark challenge would trigger an acute increase in swimming velocity. The dark phase (4 min) was divided into a startle phase (first second) and a post-startle phase (last 3 min). This test was also performed with a 48-well plate with a top-view camera in the ZebraBox, twice per day: the first test was done in pre-exposure condition of egg water, followed by a 20-min exposure period and a second test under exposure conditions of a non-lethal 1% ethanol solution

The adult fish were kept at a maximum density of 12 individuals in plastic 7.5 l tanks (1145, Tecniplast, Germany), in a zebrafish recirculation system, on a 14-h light to 10-h dark cycle. The fish were fed three times a day with dry food and frozen *Artemias.* Zebrafish eggs were obtained by random mating between sexually mature individuals. Eggs were collected, after breeding, and approximately 150 eggs were transferred to 10 cm Petri dishes (SIGMA-Aldrich L × W 55 mm × 16 mm), filled with 50 ml of egg water (0.21 g l^−1^ instant ocean sea salt and 0.0005% (v/v) methyl blue), and housed in a separate climate room. The room was maintained at a temperature of 28 °C and 30% humidity and kept under the same light–dark cycle as in the zebrafish recirculation system mentioned above. On the second day post-fertilization (2 dpf), 48 eggs were transferred to a 48-well plate (1 egg per well). The transfer was performed at 2 dpf to minimize potential physical damage to the larvae and to prevent potential handling stress to affect the behavioural results at 6, 7, and 8 dpf. The eggs and larvae were left undisturbed from 2 dpf until 6 dpf, after which the experiments commenced (see overview in Fig. 1). After the final tests at 8 dpf, the larvae were sacrificed in ice water. All procedures were approved by the ethical committee for animal experiments at Leiden University.

### Individual consistency test

We explored individual consistency in swimming activity and emergence tendency. Basal swimming activity was assessed in an open field set-up by measuring the swimming velocity (V, mm s^−1^). The experiment was conducted between approximately 9:00 and noon, corresponding to the time the zebrafish larvae are the most active (MacPhail et al. 2009; Amin et al., 2016). 48 individual larvae were transferred from the petri dish to a 48-well plate with a 3-ml Pasteur pipette. At ca. 10:00, the well plate was placed in a zebrafish observation tool, the ZebraBox (Viewpoint, Lyon, France), equipped with an infrared light source illuminating the plate from below, and an infrared sensitive camera placed at an optical height of ca. 1.5 m. The larvae were acclimatized in the ZebraBox for 1 h before tests began at 11:00. Video recordings were made during a 1-h light period (acclimation and basal activity measurements). The test was performed once a day for three consecutive days, from 6 until 8 dpf (Fig. 1a). Each zebrafish larva was subsequently transferred individually to a petri dish containing egg water (ca. 6 ml), in preparation for the emergence test. In the emergence test, we assessed the emergence time (s), which is the time it took for an individual zebrafish larva to emerge from a darkened familiar holding compartment through a narrow outlet into a well-lit and potentially dangerous open field compartment (c.f. Tudorache et al. 2014). This parameter is considered to reflect risk-taking behaviour and used as proxy for coping style in various fish species (Rey et al, 2013; Huntingford et al, 2010; Tudorache et al., 2013, 2014, 2018). The set-up used for this test consisted of two circular compartments (3.3 cm diameter × 1 cm height), one darkened and one transparent, connected by a passage tube (0.5 cm diameter × 0.6 cm length). The test was recorded by a HD camera (Panasonic HDC-SD90, Panasonic, Osaka, Japan), placed ca. 60 cm above the set-up. Recording lasted for 1 h after which the test was terminated.

The experimental procedure for the emergence test was as follows. A total number of 48 zebrafish larvae were individually transferred from the 48-well plate to a Petri dish by means of a 3-ml Pasteur pipette. After an acclimation period of 1 h, the content of the Petri dish was carefully poured into the holding compartment, with the passage tube blocked, and the novel compartment filled with 6 ml of egg water. After an additional acclimatization of 5 min, the test was started by opening the passage tube, and each individual per test was allowed to emerge from the darkened compartment into the novel compartment. At the end of the test, each zebrafish larva was transferred back into its well to undergo further testing the next day. The emergence time (ET in s), i.e. the moment of individual emergence, measured as elapsed time from the start of the test, was thereafter visually determined from the recorded 1-h video footages of the emergence tests. This entire process was repeated once a day, for 3 days, from 6 dpf until 8 dpf (Fig. 1b).

### Ethanol exposure test

The ethanol test aimed at assessing changes in activity levels caused by ethanol (EtOH) exposure at non-lethal levels (Lockwood et al. 2004; Ikeda et al. 2013; Puttonen et al. 2013; Guo et al. 2015). We were interested if the impact of ethanol exposure on behaviour would vary with the individual variation in the results of the dark challenge test (see Fig. 1). In this test, changes in activity levels are triggered by a sudden light-to-darkness transition. This dark shock typically induces an immediate anxiety-like response of elevated swimming velocity (Schnörr et al. 2012; Ellis et al. 2012; Peng et al. 2016; Luchtenburg et al. 2019), followed by gradual recovery, both to a variable extent among different individuals. This test has been used for various studies from basic behavioural research to applied drug screening (e.g. Ellis et al. 2012; Peng et al. 2016; Luchtenburg et al. 2019), including studies on alcohol effects (MacPhail et al. 2009; de Esch et al. 2012; Guo et al. 2015), and allows the exploration of individual variation in swimming tendencies and characteristics under basal and anxiety-like conditions. The swimming behaviour during these two periods were investigated in three phases: (1) the basal phase, corresponding to 1 h of the light period; (2) the startle phase, corresponding to the first second of the dark period; and (3) the post-startle phase corresponding to the last 3 min of the dark period.

The experimental procedure for the ethanol test was as follows. Zebrafish larvae of 6 dpf were transferred to a 48-well plate, placed in the ZebraBox, and a first dark challenge test was performed as described above. Subsequently, using a multipipette, 100 µl of egg water was pipetted out of each well and replaced with 100 µl of a 5% (v/v) ethanol solution, previously prepared from 95% (purity) ethanol, resulting in a non-lethal concentration of 1% ethanol. After a 20-min period for acclimation and drug uptake, the well plate was placed back into the ZebraBox, and the dark challenge test was repeated (Fig.  1c).

Swimming velocity (V, mm s^−1^) was determined as a measure for activity levels from the video footage at 25 frames per second, using EthoVision XT 6 tracking software (Noldus Information Technology B.V., Wageningen, the Netherlands). The detailed measures were nested in 1-min bins for the basal phase and in 1-s bins for the startle and post-startle phases.

### Data analyses

We collected data from two groups of *N* = 144 individuals each. One group was submitted to the basal activity and emergence test to allow for paired comparisons for consistency in time and correlation between behavioural parameters. A second independent group was used for the ethanol test. All experiments were conducted in triplicate, using three separate testing events per test, at the same time on 3 different days.

Data matrix manipulation and statistical analyses were performed using GraphPad Prism 8 (GraphPad, San Diego, California). For both tests (data on individual consistency and effects of alcohol), we checked data for normality and lognormality using a Kolmogorov–Smirnov test. We found non-normal (*p* < 0.05) distributions for all data of the individual consistency in behaviour test and of the ethanol exposure test. We subsequently used a Spearman rank correlation test for the non-normally distributed data of the individual consistency in behaviour test. In the case of comparing means of paired values for the ethanol exposure test, we used a Wilcoxon paired-signed rank test for the non-normally distributed data. Statistical significance was accepted at *p* < 0.05. All values are presented as mean ± SEM.

## Results

### Individual consistency in behaviour

**Fig. 2:**
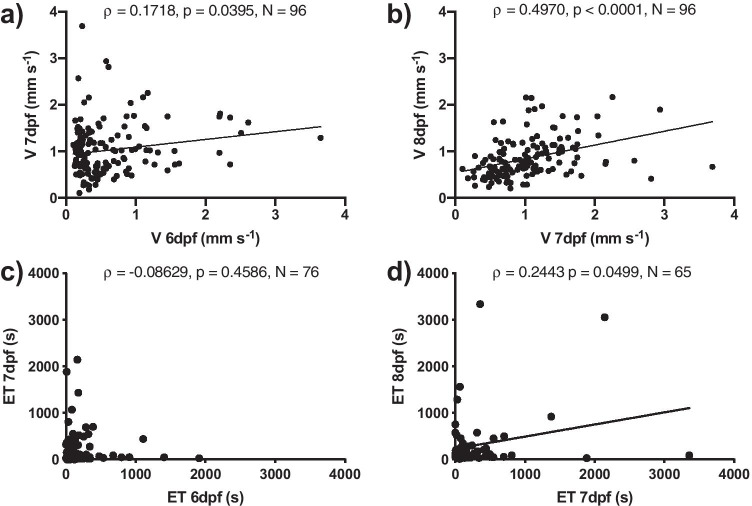
Results of the consistency tests. **a** Swimming velocity (V, mm s^−1^) of individual zebrafish larvae at 6 dpf of age compared to their swimming velocity at 7 dpf. **b** V of the same individual zebrafish larvae at 7 dpf of age compared to their swimming velocity at 8 dpf. (Spearman rank test). A line indicates a significant correlation (*p* < 0.05). V as a behavioural trait is consistent over time. **c** Emergence time (ET, s) of individual zebrafish larvae at 6 dpf of age compared to their swimming velocity at 7 dpf. **d** ET of the same individual zebrafish larvae at 7 dpf of age compared to their swimming velocity at 8 dpf (Spearman rank test). A line indicates a significant correlation (*p* < 0.05). ET as a behavioural trait is consistent over time only between 7 and 8 dpf

Swimming velocity (V, mm s^−1^) during basal activity varied along a continuous distribution from slow to fast extremes and ranged between 0.0137 to 3.688 mm s^−1^, with an average of 0.61 ± 0.04 mm s^−1^ for 6 dpf old larvae, 1.026 ± 0.0473 for 7 dpf old larvae, and 0.84 ± 0.03 mm s^−1^ for larvae at 8 dpf (*N* = 144). Individual swimming velocities were significantly correlated between 6 and 7dpf (Fig. 2a, Kolmogorov–Smirnov normality test, *p* < 0.0001; Spearman rank test, *ρ* = 0.0109, *p* = 0.0395, *N* = 144), and between 7 and 8 dpf (Fig. 2b, Kolmogorov–Smirnov normality test, *p* < 0.0001; Spearman rank test, *ρ* = 0.4970, *p* < 0.0001, *N* = 144). Most individual zebrafish larvae emerged within 15 min, and the few individuals which did not emerge within 1 h were removed from further analysis without replacement. Average emergence times, i.e. the time elapsed from the opening of the passage until observed emergence into the novel environment, were 524.2 ± 141.9 s at 6 dpf, 308.0 ± 55.46 at 7 dpf, and 336.0 ± 65.15 at 8 dpf. The emergence times of individual zebrafish larvae at 6 dpf of age were not significantly correlated to their emergence times at 7 dpf (Fig. 2c; Kolmogorov–Smirnov normality test, *p* < 0.0001; Spearman rank test, *p* = 0.4586, *N* = 76), but the emergence times at 7 dpf of age were significantly correlated to their emergence times at 8 dpf (Fig. 2d; Kolmogorov–Smirnov normality test, *p* < 0.0001; Spearman rank test, *ρ* = 0.0155; *p* = 0.0499, *N* = 65). Activity level (V) 48 and emergence time (at 8 dpf) were not significantly correlated (Spearman rank test, *ρ* = 0.0477, *p* = 0.6625, *N* = 65).

### Ethanol exposure test

**Fig. 3:**
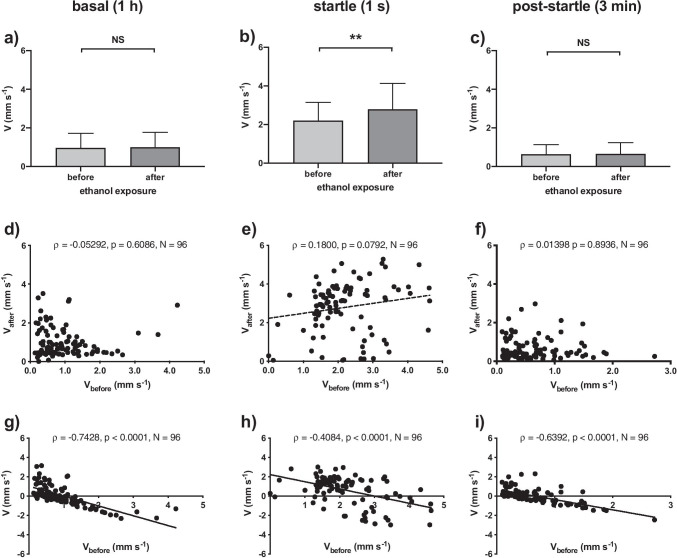
Results of the ethanol test. Swimming velocity (V, mm s^−1^) of zebrafish larvae at 6 dpf, before and after exposure to a non-lethal 1% ethanol solution in egg water. We depicted basal, startle, and post-startle phase data, respectively. **a**–**c** When considering the entire population, swimming velocity was significantly different between pre-exposure and exposure conditions only during the startle phase (**b**; Wilcoxon paired-signed rank test; ** *p* < 0.01, NS *p* > 0.05, *N* = 96), but not during basal (**a**) and post-startle phase (**c**). **d**–**f** There were no significant correlations between individual velocities in pre-exposure and exposure conditions for any of the phases (Spearman rank test). A dotted line indicates a non-significant trend (0.05 < *p* < 0.1). The consistency of inter-individual variation across days, as found in the first test, was not found within an hour in the context of ethanol exposure in this second test. **g**–**i** Activity level dependent individual variation in response yielded a significantly negative correlation between V and ΔV in all three of the phases (Spearman rank test). A line indicates a significant correlation (*p* < 0.05)

We found no significant difference between the swimming velocity in the basal phase before and after the ethanol exposure (Fig. 3a, Kolmogorov–Smirnov normality test, *p* = 0.0116; Wilcoxon paired-signed rank test, *p* = 0.9811, *N* = 96), a significant difference for the startle phase (Fig. 3b, Kolmogorov–Smirnov normality test, *p* < 0.0001; Wilcoxon paired-signed rank test, *p* = 0.0002, *N* = 96), and again no significant difference for the post-startle phase (Fig. 3c, Kolmogorov–Smirnov normality test, *p* < 0.0001; Wilcoxon paired-signed rank test, *p* = 0.8985, *N* = 96). Individual swimming velocities were also not significantly correlated between pre-exposure and exposure conditions in any of the phases (Fig. 3d–f, Kolmogorov–Smirnov normality test, *p* < 0.0001; Spearman rank test, *p*_basal_ = 0.6086, *p*_startle_ = 0.0792, *p*_post-startle_ = 0.8936, *N* = 96). The reason for this was that we found activity level dependent individual variation with a significantly negative correlation between the pre-exposure swimming velocity and the difference between pre-exposure and exposure values, for each of the three phases (ΔV, mm s^−1^, Fig. 3g–i, Kolmogorov–Smirnov normality test, *p* < 0.0001; Spearman rank test, *p*_basal_ > 0.0001, *ρ* =  − 0.7428, *p*_startle_ > 0.0001, *ρ* =  − 0.4084, *p*_post-startle_ > 0.0001, *ρ* =  − 0.6392, *N* = 96). Low basal activity individuals increased swimming velocity in response to the ethanol treatment, while high basal activity larvae decreased their speed, and larvae of intermediate activity level showed little response to the ethanol exposure.

## Discussion

We used zebrafish larvae to explore individual consistency in behaviour and to investigate the effect of consistent individual variation on the effect of alcohol. We found that (1) individual variation in activity level was consistent over time between larvae of 6 dpf and 7 dpf, and larvae of 7 dpf and 8 dpf, and that (2) individual variation in emergence time was not yet correlated for larvae of 6 dpf and 7 dpf, but significantly correlated for larvae of 7 dpf and 8 dpf. We found (3) no indication that consistent individual variation in activity level was correlated to consistent individual variation in emergence time (tested at 8 dpf). Individual zebrafish larvae exhibited a highly variable behavioural response to the ethanol treatment: (4) individual consistency in time in activity level did not show up again, as low- and high-activity zebrafish larvae of 6 dpf responded diametrically opposite to a non-lethal 1% ethanol concentration. The variable impact was reflected in a significantly negative correlation between the basal activity level and the velocity change induced by the ethanol treatment. Relatively slow individuals raised their swimming velocity as expected, but relatively fast individuals slowed down from pre-exposure to exposure conditions.

### Activity level as proxy for coping style

The consistency in inter-individual differences in activity level over time, between subsequent days in early development (6–7 dpf and 7–8 dpf), suggested that this is a practical measure for coping style in behavioural screening studies. However, despite the fact that activity level may be related to other traits belonging to particular behavioural and physiological phenotypes in zebrafish (Egan et al. 2009; Oswald et al 2012; Tran and Gerlai 2013a; Tudorache et al. 2013; Tran et al. 2016a), we did not find a correlation between the variation in emergence time and the individual variation in activity for our zebrafish larvae. This is in contrast to an earlier study in which we used the same emergence test set-up and assessed activity level (Tudorache et al. 2014).

The context of the previous and current emergence tests differed in an important way, which may explain the lack of the correlation with activity level in the current study. Tudorache et al. (2014) conducted the emergence test in a social context with a group of 10 larvae, while the current emergence test concerned a solo test with a replicate set of single larvae. Social context has been reported before to undermine repeatability of personality traits in various fish species, as variability among individuals tend to be higher when individuals are not affected by shoal mates (Gómez-Laplaza and Morgan 1991; Magnhagen and Bunnefeld 2009; Jolles et al. 2016). The maximum emergence time also differed considerably in our studies, with 331 s for the group emergence test (Tudorache et al. 2014) and 3514 s for the single emergence test in the current study. This tenfold increase could be interpreted as an increase in risk-aversiveness when zebrafish larvae are tested by themselves instead of in a group (Wright et al. 2003; Al-Imari and Gerlai 2008; Miller and Gerlai 2011). The discrepancy in methods probably means that individual variation in risk-taking behaviour was measured over a different range of the scale, and apparently matching less well with the range determining general swimming activity.

We believe that more studies are warranted to explore variation in emergence time between social and solo test conditions. In adult individuals of other fish species, social conditions have also been shown to affect behavioural tendencies, with individuals that were kept in a group instead of solo prior to testing being more active and explorative (Gómez-Laplaza and Morgan 1991; Jolles et al. 2014). If the context is compared in one and the same experiment, unlike our current comparison between two different studies (Tudorache et al. 2014 and the current study), it is also easier to exclude potentially confounding variables for the current speculation, such as subtle variation in light or handling conditions, which may vary per room and experimenter and which may affect relative stress levels of the larvae in the test. Across-context consistency in different behaviours has been reported repeatedly for adults (Egan et al. 2009; Oswald et al 2012; Tran and Gerlai 2013a), also at Leiden University (Tudorache 2013; 2018), but remains an avenue for further exploration in zebrafish larvae. Nevertheless, the inter-individual consistency in activity level for larvae, already from 6 dpf, was a repeatable finding and concerns an important factor to take into account for the interpretation of data from behavioural screening studies (Egan et al. 2009; Araujo-Silva et al. 2018; 2020; Demin et al. 2019).

### Activity level dependent ethanol impact

We found significant variation in the scale and direction of ethanol impact among individuals of the same laboratory strain of zebrafish, which was dependent on activity level, measured as swimming velocity. General activity levels were found to increase from 6 to 7 dpf and decreased from 7 to 8 dpf, possibly due to a cyclical swimming mode being replaced by an undulatory swimming mode with age, resulting in a temporarily reduced efficiency of tail movement and therefore speed (Müller and van Leeuwen 2004). When analysing the average data of the entire population, we found no effect of ethanol on activity level in the basal and post-startle phase, and only a small effect in the startle phase. The analysis of individual variation in velocity change revealed the underlying reason for a lack in consistency between activity level before and after ethanol exposure: slow individuals raised activity level, fast individuals dropped activity level, and intermediate individuals did not change at all. This result was consistent across the basal, startle, and post-startle phases and revealed a dynamic individual trait related to basal activity level. Our results therefore show that the presence of such a personality trait in a plastic response can yield diametrically opposite reactions from individuals at both ends of the behavioural continuum on the low to high activity axis. This suggests that depicting and testing group averages may obscure underlying mechanisms, as responses may be different for different individuals (MacKenzie et al. 2009; Tudorache et al. 2018; Demin et al. 2019).

The underlying mechanism for the opposite impact of 1% ethanol exposure in our study for individuals varying in activity level is unclear, but we can make some tentative suggestions. It has become clear from several studies that ethanol affects dopamine and serotonin neurotransmitter systems as well as cortisol release through activation of the hypothalamic-pituitary-interrenal axis (Gerlai et al. 2009; Rosemberg et al. 2012; Tran et al. 2015; 2016a). Consequently, the typical biphasic response is not only reflected in a rise and fall in activity level over time, but also in various anxiolytic and anxiogenic effects on behaviours such as swimming velocity, turning rate, freezing tendency, swimming depth, explorative behaviour, and social shoaling tendencies. As individual coping styles also vary in the responsiveness of these very same physiological systems and behavioural read-outs (Egan et al. 2009; Tran et al. 2016b; Rey et al. 2013; Tudorache et al. 2018), it should be no surprise to find interactive effects through direct neurophysiological processes (c.f. Araujo-Silva et al. 2018, 2020; Leite-Ferreira et al. 2019).

It may also be that there is an indirect explanation for the activity-dependent impact on effects of alcohol. Slow individuals also have a slow metabolism and therefore slow ethanol uptake, which could give them a delay relative to the fast individuals in going through the time course of the typical biphasic response pattern (Gerlai et al. 2006; Tran and Gerlai 2013b; Tsang et al. 2019). The gradual and steady rise in ethanol uptake into the brain over time (Gerlai et al. 2009; Rosemberg et al. 2012; Tran et al. 2015; Guo et al. 2015) may just have varying time courses for fish with different coping styles. The individuals at the low activity level end of the range may still experience stimulating effects, while kinematically and metabolically fast individuals already progress into the subsequent destructive and sedative phases.

Another explanation for the opposite response of coping style extremes is the possible effect of ethanol on oxygen concentration in the water and temperature. It is known that ethanol reduces bacterial load of the water and could therefore lead to a reduction in the bacterial oxygen consumption rate and a reduced metabolic waste production. Furthermore, ethanol evaporates, which can reduce the temperature of the water, which may reduce the metabolic rate of the fish and increase the oxygen diffusion rate. Consequently, the differences of larval activity levels during the exposure phase could reflect the individual’s capability to respond to the ethanol-induced changes in oxygen concentration and temperature, rather than an effect of ethanol on the central nervous system. Irrespective of the underlying physiological process, the activity level-dependent pattern of response to ethanol exposure remains a factor better taken into account.

### Taking coping style into account

The diametrically opposite response patterns to ethanol among individual larvae of consistently divergent behavioural phenotypes clearly showed the importance of taking coping styles into account when interpreting behavioural screening studies. Many studies on other fish species have investigated the natural range of coping styles and have repeatedly shown the existence of behavioural variation along bold-shy axes, often yielding distinct behavioural phenotypes (Øverli et al. 2007; Toms et al. 2010; Conrad et al. 2011). Proactive individuals are typically more bold, explorative, and aggressive, with a high level of locomotor activity and metabolic rate, while the opposite is found in reactive fish. MacKenzie et al. (2009) already provided a nice example in carp (*Cyprinus carpio*), in which this kind of variation in coping styles affected the outcome of a laboratory study, which was critical for the mechanistic explanation. After determining the coping style of carp in an emergence test, they exposed them to a bacterial lipopolysaccharide challenge, causing an inflammatory response, and measured the transcription of genes related to the immune system, in the brains of individual fish. Shifts in gene expression pattern were highly variable and differed for the whole group, compared to subsets of proactive and reactive fish. Carp of different coping styles differed already in baseline expression of a number of genes and showed diametrically opposite responses to the challenge for 80% of the genes investigated. MacKenzie et al. (2009) argued that incorporating coping style as an explanatory variable can account for unexplained variation that is common in gene expression studies. We support the argument of MacKenzie et al. (2009) and would like to extend this to ethanol impact and possibly other toxicological and pharmacological studies (c.f. Demin et al. 2019).

## Conclusion

In most biological and pharmacological studies, there is a well-established awareness about the need to control and minimize variation among individuals within groups by using, for example, specific genetic backgrounds. Nevertheless, it is still common to detect significant levels of individual variation, which might be caused by interactions between the coping style of the individual and the specific treatment of study. Our case study on ethanol impact on zebrafish larvae adds a clear case for why we believe it is important to better understand the concept of coping styles and to take them into account during statistical analyses. We do not yet suggest activity level to be an ideal proxy for coping style, as further ties to other traits of potential behavioural syndromes still need to be investigated for zebrafish larvae. However, our results clearly show that taking basal activity level into account can improve power for statistical testing in any behavioural screening study.
